# How and How Much Molecular Conformation Affects Electronic Circular Dichroism: The Case of 1,1-Diarylcarbinols

**DOI:** 10.3390/molecules23010128

**Published:** 2018-01-09

**Authors:** Daniele Padula, Gennaro Pescitelli

**Affiliations:** 1 Department of Chemistry, University of Liverpool, Liverpool L69 7ZD, UK; dpadula@liverpool.ac.uk; 2 Department of Chemistry and Industrial Chemistry, University of Pisa, Via G. Moruzzi 13, 56124 Pisa, Italy

**Keywords:** stereochemistry, conformational analysis, absolute configuration, electronic circular dichroism calculations, vibronic circular dichroism, benzene sector rules

## Abstract

Chiroptical spectra such as electronic circular dichroism (ECD) are said to be much more sensitive to conformation than their non-chiroptical counterparts, however, it is difficult to demonstrate such a common notion in a clear-cut way. We run DFT and TDDFT calculations on two closely related 1,1-diarylmethanols which show mirror-image ECD spectra for the same absolute configuration. We demonstrate that the main reason for the different chiroptical response of the two compounds lies in different conformational ensembles, caused by a single hydrogen-to-methyl substitution. We conclude that two compounds, having the same configuration but different conformation, may exhibit mirror-image ECD signals, stressing the importance and impact of conformational factors on ECD spectra.

## 1. Introduction

Electronic circular dichroism (ECD), the differential absorption of left- vs. right-circularly polarized light in the domain of electronic transitions, is the most popular and widespread chiroptical technique used for the characterization of chiral non-racemic samples [1]. ECD is the chiroptical counterpart of UV-Vis absorption spectroscopy, in the same way all other chiroptical techniques, such as vibrational CD (VCD), optical rotation dispersion (ORD), circularly polarized luminescence (CPL), etc., have their non-chiroptical counterparts, respectively infrared (IR) spectroscopy, refractive index dispersion, luminescence emission spectroscopy, and so on [2]. The main advantage of chiroptical spectroscopies over their non-chiroptical analogues is obviously the fact that the former ones are sensitive to the molecular and supramolecular chirality. In fact, each band in a chiroptical spectrum may have either a positive or negative sign, which is always opposite for the two enantiomers of the same substance. Hence, the main application of these spectroscopies is in the assignment of absolute configuration [1,2]. However, it is often said that chiroptical spectroscopies are also more sensitive to the molecular conformation than their non-chiroptical analogues [3]. This means that, when assigning absolute configurations, the molecular conformation must be studied as well; but, also, that (especially) ECD and VCD lend themselves as tools for studying molecular and supramolecular conformation [1,4,5]. In more fundamental terms, ECD and VCD spectra, including their sign, are determined not only by the molecular *configuration*, but also by the molecular *conformation*, and more generally by a combination thereof.

The dependence of ECD on conformational factors has been discussed in several instances, especially in combination with ECD quantum-chemical calculations [6,7,8,9,10,11,12,13,14,15,16,17,18,19]. In fact, it is easy to demonstrate “in silico” that different conformations of the same molecule exhibit different chiroptical responses. It is of course much more difficult to get a valid experimental proof of the same phenomenon. To this end, one must resort to more or less complex systems such as conformation-dependent chiroptical switches [20,21,22] or achiral compounds forming chiral crystals by spontaneous resolution [23]. Only the use of emerging or niche techniques such as two-photon ionization CD and photoelectron CD may offer an actual snapshot of molecules entrapped in a specific conformation [24,25].

**Scheme 1:**
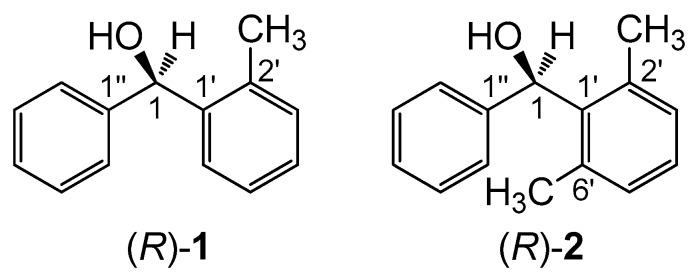
Chemical structures of (*R*)-(2-methylphenyl)(phenyl)methanol (*R*)-(**1**) and (*R*)-(2,6-dimethylphenyl)(phenyl)methanol (*R*)-(**2**).

**Figure 1:**
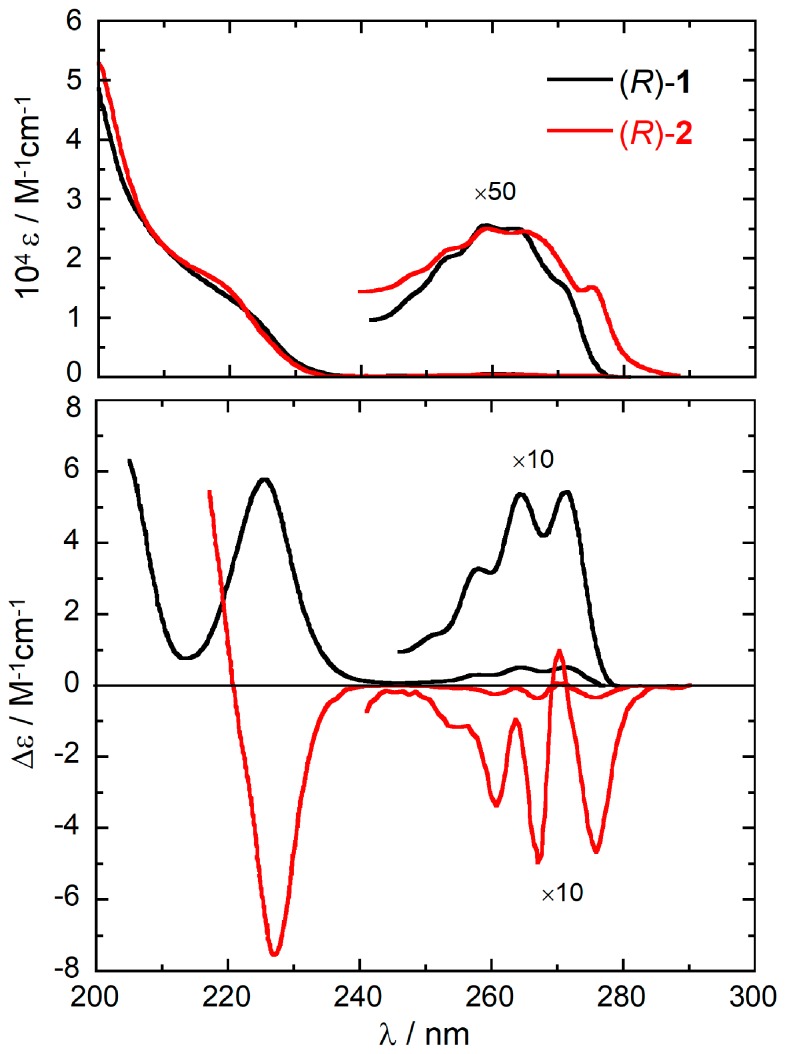
Experimental UV (**top**) and ECD (**bottom**) spectra of (*R*)-**1** (black lines) and (*R*)-**2** (red lines) recorded in ethanol. Reproduced from Ref. [28] with permission of Taylor & Francis Ltd., www.tandfonline.com.

In a much simpler but very clever and didactic way, a good means of demonstrating the impact of molecular conformation on ECD spectra would consist in finding a pair of compounds with a very similar skeleton and consistent absolute configuration, but showing very different ECD spectra, possibly even the mirror image of each other. A very nice example of this kind was reported in 1997 by Sandström and co-workers concerning two analogous spiro compounds which would show mirror-image ECD spectra for the same configuration at the spiro center, due only to a different cycle size (5- vs. 6-membered) [26]. Unfortunately, we showed recently that this example is faulty, because one absolute configuration was wrongly assigned [27]. A second good example is offered by the two compounds investigated here, (2-methylphenyl)(phenyl)methanol (**1**, Scheme 1) and (2,6-dimethylphenyl)(phenyl)methanol (**2**). These two alcohols were reported in 2002 by Harada, Pirkle and coworkers [28]. They were prepared in enantiopure form and their absolute configuration was unambiguously assigned by X-ray, taking advantage of a chiral derivatizing agent based on camphorsultam dichlorophthalic acid [28,29]. The two compounds differ only for one methyl group attached at position 6’ of one phenyl ring (Scheme 1). For the same absolute configuration (*R*) of the carbinol carbon, the two compounds display almost mirror-image ECD spectra over the whole available range 210–300 nm (Figure 1) [28,30]. The authors stressed that the empirical comparison between the ECD spectra of **1** and **2** would lead to an incorrect assignment of one absolute configuration. Such empirical comparisons were still quite popular at the time of the original publication, while they are heavily discouraged nowadays [1,2]. The authors postponed to a future study the reason why compounds (*R*)-(+)-**1** and (*R*)-(−)-**2** show opposite ECD spectra. However, although the original paper has been quoted several times as a warning against empirical spectral comparisons [27,30,31,32,33,34], an interpretation of the observed phenomenon has not been provided yet, to the best of our knowledge. 

In the present paper, we report the results of a computational study on **1** and **2** by means of density functional theory (DFT) calculations. Our approach was able to reproduce the experimental spectra of (*R*)-(+)-**1** and (*R*)-(−)-**2**, including the vibrational fine structure (vibronic ECD calculations on **2** have been reported previously) [35]. We shall see that the difference in the ECD spectra of (*R*)-**1** and (*R*)-**2** is mainly a consequence of different conformational ensembles, stressing the impact of conformational factors on the appearance of ECD spectra. In conclusion, we shall demonstrate that two simple compounds **1** and **2** with the same absolute *configuration* at the chiral centers display almost mirror image ECD spectra because of their different *conformation*.

## 2. Results

### 2.1. Experimental and Calculated ECD Spectra

The absorption and ECD spectra of compounds (*R*)-(+)-**1** and (*R*)-(–)-**2** are reproduced in Figure 1. They show the typical bands of substituted benzene chromophore [36], namely: (a) the ^1^L_b_ band between 250–275 nm, with low intensity and characteristic vibrational structure [37]; (b) the ^1^L_a_ band between 210–250 nm, with relatively higher intensity both in the UV and ECD spectra. Unfortunately, the high energy region corresponding to the ^1^B_b_ band is not covered [28,30]. While the UV spectra of the two compounds are very similar to each other, the ECD spectra look almost the mirror image of each other over the whole available range.

**Figure 2:**
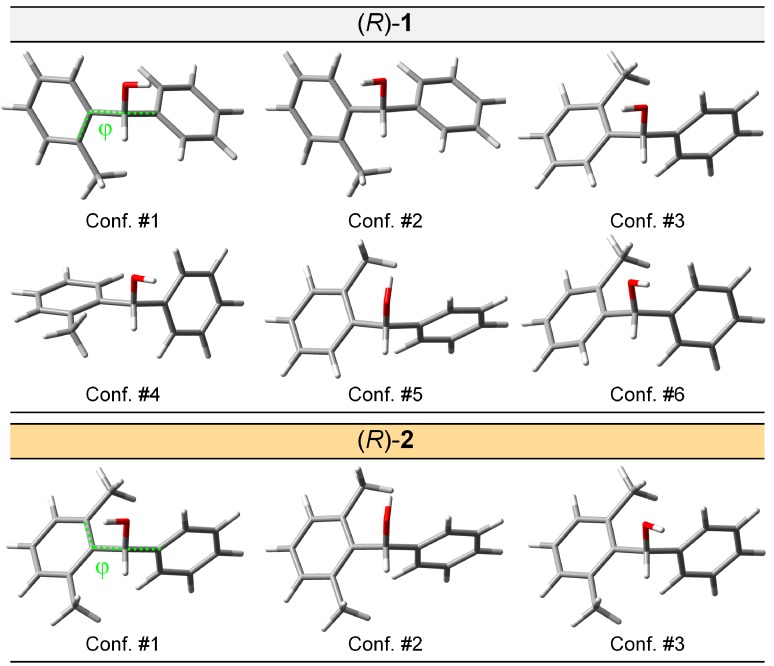
Low-energy conformers of compounds (*R*)-**1** and (*R*)-**2** calculated at ωB97X-D/def2-TZVP/PCM level. The dotted lines indicate the dihedral angle defined by carbon atoms 1″–1–1′–2′.

To simulate the ECD spectra of **1** and **2**, we used a consolidated computational procedure [38,39]. First, we investigated the conformational energy surface by means of conformational searches with the Monte Carlo algorithm implemented in Spartan’16 [40] using the Merck Molecular Force Field (MMFF). MMFF torsional energy scans were also run on the relevant torsional modes to assure that all possible conformers had been detected. All MMFF structures were optimized with DFT at ωB97X-D/def2-TZVP level including a polarizable continuum solvent model (PCM) for ethanol [41]. We found six low-energy conformers for (*R*)-**1** and three low-energy conformers for (*R*)-**2**, described in Table 1 and Figure 2. The accuracy of relative energy estimates was checked for (*R*)-**2** with several other computational methods, including the augmentation of the basis set, the use of a double hybrid functional (B2PLYP) or coupled-cluster calculations (CCSD), and a different solvation model, as reported elsewhere [35]. Since no significant differences were found with respect to ωB97X-D/def2-TZVP/PCM, this latter method was used thoroughly in the present work.

ECD calculations were run on compounds (*R*)-**1** and (*R*)-**2** with the time-dependent DFT (TDDFT) method [39] at the ωB97X-D/def2-TZVP/PCM level. It is well known that excited-state TDDFT calculations of benzene derivatives are heavily affected by the nature of the density functional [42,43,44,45]. The present choice was based on our previous experience with this class of compounds [35,46,47], as well as on the general good performance of ωB97X-D functional [48] in the prediction of chiroptical spectra [38]. Consistent results were obtained at the CAM-B3LYP/def2-TZVP/PCM level. The ECD spectra calculated for each conformer were averaged using Boltzmann factors evaluated at 300 K from internal energies. We are aware that different averaging strategies which take into account low-energy internal motions and/or based on free energies, may lead to different and possibly more accurate average ECD spectra [38]. However, a few more sophisticated strategies were tested for compound **2** without leading to significantly different or improved results with respect to the straightforward Boltzmann averaging employed here [35].

**Figure 3:**
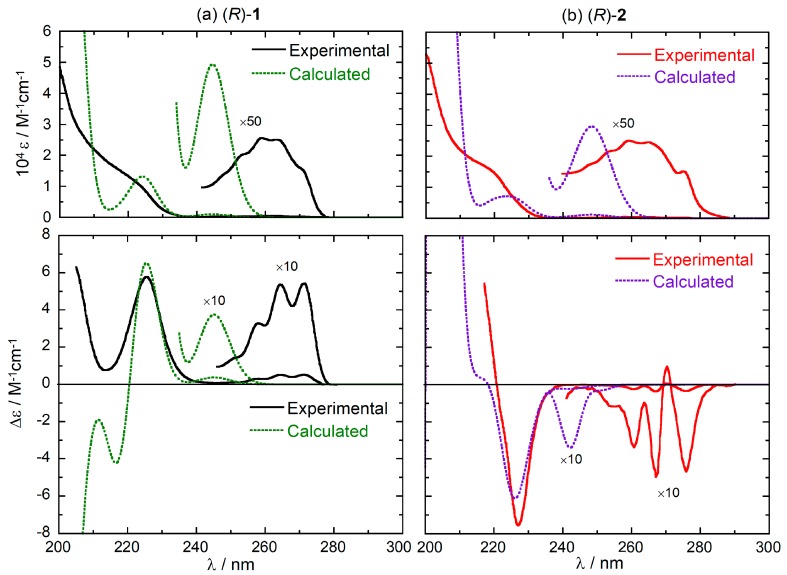
Comparison between the experimental and weighted-average calculated UV (**top**) and ECD (**bottom**) spectra of (*R*)-**1** (panel **a**) and (*R*)-**2** (panel **b**). Calculated spectra plotted as sums of Gaussians with 0.15 eV exponential band-width, shifted by +15 nm and scaled for better comparison. Experimental spectra reproduced from Ref. [28] with permission of Taylor & Francis Ltd., www.tandfonline.com.

The average calculated UV and ECD spectra for (*R*)-**1** and (*R*)-**2** are reported in Figure 3 along with the experimental spectra, for an immediate comparison (full calculated spectra including the ^1^B_b_-band region are shown in Figure S1, Supplementary Material). The calculations suffer from an underestimation of the energy difference between ^1^L_a_ and ^1^L_b_ bands, which is related to the functional employed [42,43,44,45]. Most importantly, however, they predict the correct sign for all ECD bands in the ^1^L_a_ and ^1^L_b_ regions for both compounds. This is a prerequisite as we wish to use (TD)DFT calculations to understand the origin of the different ECD spectra of (*R*)-**1** and (*R*)-**2**. To reproduce the vibrational pattern seen in the experimental spectra, electronic CD calculations are of course not sufficient and vibronic ECD calculations must be employed, as described in the next subsection.

### 2.2. Vibronic ECD Calculations (^1^L_b_ Band)

The vibrational progression seen in the experimental UV and ECD spectra of compounds **1** and **2** (Figure 1) is typical for the electric-dipole forbidden ^1^L_b_ transition of simple aromatic molecules containing substituted phenyl rings [47,49,50]. We studied previously this aspect for a few benzene derivatives, including the present compound **2** [35,47]. We refer the reader to the quoted papers for the details of the computational approach, the analysis of the vibronic coupling mechanism and the ECD spectra obtained thereof. Here we applied the Vertical Hessian (VH) model, by computing explicitly the Hessian of the first two excited states for each molecule [51]. The VH model takes into account both the effect of frequency changes between the ground and excited states, and Duschinsky mixings. A development version of the FCclasses code [52] was employed in vibronic calculations; the Franck-Condon terms were estimated at 0 K, using internal coordinates (the so-called VHint model) [53] and including the first two transitions for each compound (one ^1^L_b_-type transition localized on each ring). The calculations were run on all low-energy conformers of **1** and **2** and averaged similarly to ECD spectra.

**Figure 4:**
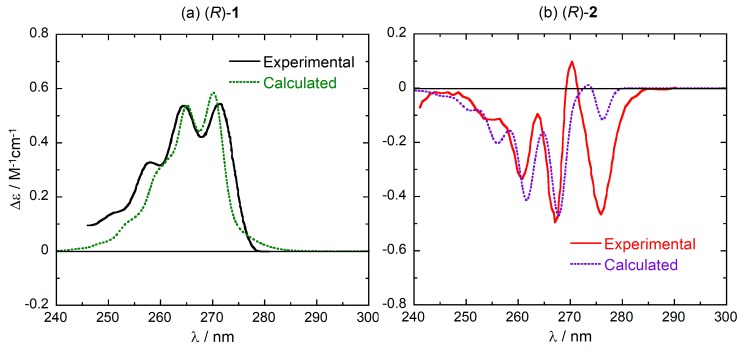
Comparison between the experimental and weighted-average calculated vibronic ECD spectra of (*R*)-**1** (panel **a**) and (*R*)-**2** (panel **b**) in the ^1^L_b_ region. Calculated spectra plotted as sums of Gaussians with 0.025 eV exponential band-width, shifted by +30 nm and scaled by a factor 5 for better comparison. Experimental spectra reproduced from Ref. [28] with permission of Taylor & Francis Ltd., www.tandfonline.com.

Calculated vibronic ECD spectra in the ^1^L_b_ region for compounds (*R*)-**1** and (*R*)-**2** are shown in Figure 4 and compared with the experimental spectra (see Figure S2, Supplementary Material, for the corresponding absorption spectra). The calculations are able to reproduce the pattern of vibronic ECD bands reasonably well. In particular, the dominant sign of the vibronic progression (positive for (*R*)-**1** and negative for (*R*)-**2**) is reproduced, and it is consistent with that of purely electronic CD calculations discussed above (Figure 3). Some minor details of the spectra are not captured by the calculations, for example the intensity of the first two peaks found for (*R*)-**2** at 270 and 276 nm. A complete treatment of these issues falls outside the scope of the present paper, and has been already discussed elsewhere for compound **2** [35]. Moreover, they do not affect the following discussion which aims at elucidating the conformational dependence of the ECD spectra of (*R*)-**1** and (*R*)-**2**.

Similarly to what was previously observed for compound **2** [35], the vibronic transitions responsible for the observed progressions are fundamental bands of modes with frequencies of 1050–1250 cm^−1^, involving combined C–H bendings and C–C stretchings of the phenyl rings (ring breathing). For the second excited state, moreover, significant progressions are also seen along low-frequency bending modes of both phenyl rings. In summary, the apparent vibronic progression of 1000–1200 cm^−1^ is due to the combination of fundamentals of the mentioned high-frequency modes, and simultaneous excitations of high- and low-frequency modes.

## 3. Discussion

In this section, we will try to identify the major source of the differences seen in the ECD spectra of (*R*)-**1** and (*R*)-**2**, in particular the almost mirror-image relationship between their ECD spectra. Because of the close structural relationship between the two compounds, it was anticipated that the sign reversal might occur for two possible reasons: (a) a change in the direction of the transition dipole moment due to the so-called substituent spectroscopic moment [54,55]; (b) a different conformational situation. The first reason would emphasize the small but non-negligible spectroscopic moment of the methyl group attached at C-6′ in **2** with respect to the corresponding H-6′ in **1**. This kind of effect, namely a rotation of transition moment directions due to the aromatic ring substituents, has been invoked a few times to justify unexpected ECD properties, including sign reversal [56,57]. However, in the present case, a major role is in fact played by conformational factors. As reported in Section 2.1, compounds **1** and **2** vary substantially in their conformational ensemble. Because of the 2′,6′-dimethyl substitution, compound **2** is relatively rigid. The three possible conformers differ practically only in the rotation of the OH group, while the reciprocal arrangement of the aromatic rings is consistent. This is demonstrated by the torsion angle *ϕ* (defined by carbon atoms 1″–1–1′–2′) measured on DFT-optimized geometries, which attains values between −60 and −70 deg for all conformers of **2** (Table 1 and Figure 2). As a consequence, the ECD spectra calculated for the various conformers are very similar to each other (Figure S4, Supplementary Material). The ^1^L_a_ band has a negative sign and similar intensity for all conformers; the ^1^L_b_ band is weak and negative for conformers #1 and #3 and, as the only exception, bisignate for conformer #2. The situation does not change much by including vibronic effects [35].

**Figure 5:**
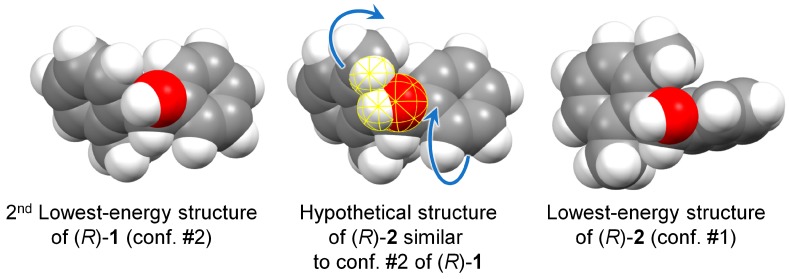
Space-filling models (90% Van der Waals radii) of the lowest-energy conformer of (*R*)-**2** (**right**) and the second lowest-energy conformer of (*R*)-**1** (**left**), and hypothetical structure of (*R*)-**2** in the same conformation of (*R*)-**1** (**middle**). In this latter, the atoms highlighted in yellow have short contacts (distance < sum of VdW radii), which are relieved by means of the rotations indicated by the blue arrows (leading to the lowest-energy structure on the **right**).

With respect to compound **2**, compound **1** is relatively more flexible: the fact that the C-6′ *ortho* carbon is not substituted, allows a further degree of conformational freedom around the C-1/C-1′ bond. In fact, the dihedral angle *ϕ* now varies a lot among the various conformers, assuming either positive or negative values (Table 1 and Figure 2). As a consequence, the calculated ECD spectra vary a lot among the various conformers (Figure S3, Supplementary Material). The decisive difference with respect to **2** is, however, the relative arrangement of the aromatic rings found in the dominant conformation. The conformational ensemble found for **1** includes three conformers (#3, #5 and #6, Figure 2) which roughly correspond to those found for **2** (#1, #2 and #3, respectively) as for the orientation of the aromatic rings (see negative values of angle *ϕ* in Table 1) and OH group. However, they account for only 18.9% of the overall population; the two most stable conformers of **1** (#1 and #2), accounting for 73.4% population, feature a different orientation of the aromatic rings (with positive values of angle *ϕ*, Table 1). The situation is well described by using space-filling models to account for steric effects. In Figure 5, we compare the lowest energy conformer of **2** with the 2nd-lowest energy conformer of **1** (the choice is dictated by the consistent orientation of the OH group in the two structures). The favored conformation observed for compound **1** cannot be assumed by compound **2**, because it would imply a steric clash between the 6’-methyl and the OH group. To avoid such contact, the substituted phenyl ring in **2** is rotated ≈40 deg clockwise around the C-1″/C-1 bond (the movement is depicted by the top blue curved arrow in Figure 5). At that point, the same methyl group would collapse with the *ortho*-hydrogen of the phenyl ring, which is avoided by rotating this latter ≈90 deg clockwise around the C-1′/C-1 bond (bottom blue curved arrow).

**Figure 6:**
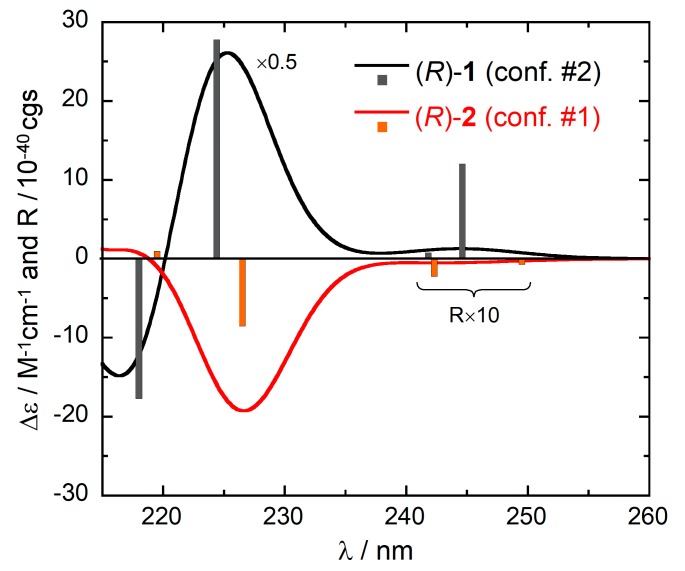
Comparison between the and calculated ECD spectra of (*R*)-**1** (conformer #2) and (*R*)-**2** (conformer #1). Plotting parameters as in Figure 2. The vertical bars represent the calculated rotational strengths R, in 10^−40^ cgs units (scaled by a factor 2.5 to fit the vertical axis).

The effect of the different conformation of **1** and **2** on the ECD spectra is displayed in Figure 6, which shows the ECD spectra calculated in the ^1^L_a_/^1^L_b_ regions for the two structures just discussed. For (*R*)-**1** (conformer #2), the first three calculated transitions have positive rotational strengths, while for (*R*)-**2** (conformer #1), the corresponding transitions have negative rotational strengths. These are the same signs found for the average calculated spectra, as well as for the experimental ones (Figure 3). Thus, the different *conformations* are truly responsible for almost mirror-image ECD spectra, despite the same absolute *configurations*.

To gain insight into the effective structure-to-spectra relationship, one must focus on a specific mechanism of optical activity [58,59]. At first look, one may expect for compounds **1** and **2** that the presence of two aromatic rings would yield an exciton coupling interaction, that is, a “dynamic” coupling between the chromophores. This is however not the case for the ^1^L_b_ bands, because of their electric-dipole forbidden character, leading to negligible exciton-coupled rotational strengths [60]. Our expectation is confirmed by the fact that the two rotational strengths in the ^1^L_b_ region have the same sign for most low-energy conformers of **1** and **2** (see for example Figure 6). An explicit calculation of the coupling potential between the ^1^L_b_ transition densities led to values reaching at most 30 cm^−1^ for some conformer of **1**. Therefore, for the ^1^L_b_ bands, we must look at a “static” mechanism of optical activity, whereby the transition of each aromatic chromophore is perturbed by the fragments in its surrounding, which act as perturbers [58,59]. The situation for the ^1^L_a_ band is more complex because both static and dynamic mechanisms are likely to concur, however, the following reasoning applies similarly well at least to the static component.

**Figure 7:**
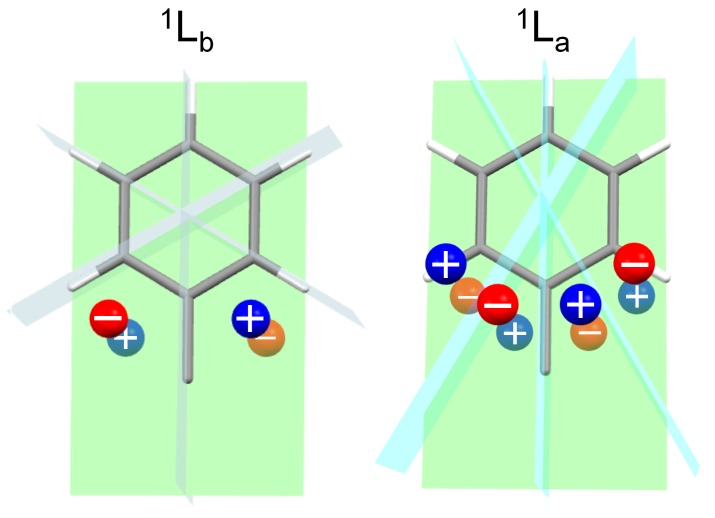
Sector rules for ^1^L_a_ and ^1^L_b_ ECD bands of monosubstituted benzene. The nodal planes (green: ring plane; light blue or cyan: perpendicular to ring plane) divide the space into sectors. A substituent, or a piece of a substituent, is viewed as a perturber placed in each sector and is represented by a sphere. Its contribution to the ECD band has the sign drawn on the spheres (blue, positive; red, negative). In the presence of further substituents on the ring (e.g., methyl groups attached at 2′ and 6′), the vertical nodal planes may be rotated around the vertical axis by an angle proportional to the substituent spectroscopic moment.

To illustrate how the static mechanism operates on the present compounds, we will make use of sector rules. Semi-empirical sector rules have been very popular in the past as tools to predict ECD spectra of various molecules [61,62]. Their use is discouraged today in favor of non-empirical methods of analysis, however, the basic theory behind sector rules remains valid and offers a simple and effective way for understanding the relation between molecular structure and ECD signals. Any sector rule is valid for a specific electronic excitation, and it is based on three conceptual steps [1]: (1) dissect the molecule into chromophore(s) and perturber(s); (2) partition the space around the chromophore into sectors, delimited by nodal planes allied with the symmetry of the electronic excitation; (3) look at the occupation of each sector by the various perturbers, possibly in reference with their relative polarizability. Two sector rules exist for predicting the sign of ^1^L_a_ and ^1^L_b_ ECD bands of simple benzene derivatives [63,64,65], illustrated in Figure 7. 

**Figure 8:**
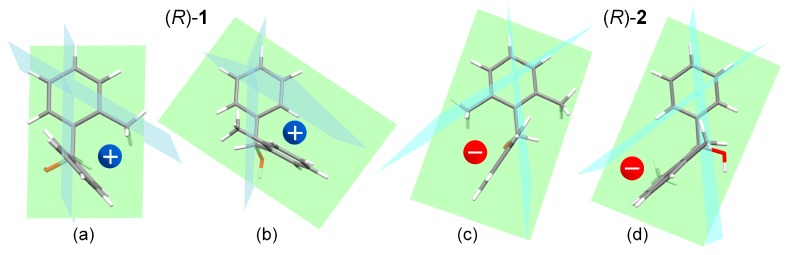
Application of the sector rule for ^1^L_b_ ECD band to (*R*)-**1** (conformer #2) and (*R*)-**2** (conformer #1). The colored spheres indicate the dominant contribution of one benzene acting as a perturber for the other ring. Structure (**a**), sector contribution of the phenyl ring on the 2-methylphenyl ring in (*R*)-**1**; (**b**), contribution of 2-methylphenyl on phenyl in (*R*)-**1**; (**c**), contribution of phenyl on 2,6-dimethylphenyl in (*R*)-**2**; (**d**), contribution of 2,6-dimethylphenyl on phenyl in (*R*)-**2**.

Because compounds **1** and **2** contain two different benzene rings, to apply the sector rules we must consider one benzene ring at a time, and look at the other ring as a perturber of the first one. The situation for the ^1^L_b_ ECD band is depicted in Figure 8. For compound (*R*)-**1** (conformer #2), the two structures on the left show that the sign of the perturbation exerted by the phenyl ring on the 2-methylphenyl (structure a) is positive, as it is that of the perturbation exerted by the 2-methylphenyl on the phenyl (structure b). For compound (*R*)-**2** (conformer #1), the situation is exactly the opposite: the perturbations exerted by the phenyl ring on the 2,6-methylphenyl (structure c), and by the 2,6-methylphenyl on the phenyl (structure d), are both negative. It can be argued that the same situation occurs for the ^1^L_a_ band, namely, the perturbation of each aromatic ring on the second one makes a positive contribution for (*R*)-**1** and a negative one for (*R*)-**2**. In this case, the static mechanism is however also accompanied by exciton coupling.

The above analysis proves that the mirror-image appearance of the ECD spectra of compounds **1** and **2** in the ^1^L_a_/^1^L_b_ regions may be rationalized taking into account the different conformation assumed by the two compounds. In particular, the two aromatic rings perturb each other differently in the two compounds, because of a different reciprocal orientation. The sign of the contribution to the ECD band, predicted by means of sector rules, is opposite for (*R*)-**1** and (*R*)-**2**. Whether the static mechanism of optical activity is dominant or not, our analysis demonstrates that the different conformation has a major impact on the ECD spectra of the two compounds.

## 4. Materials and Methods

Conformational searches were run with Spartan’16 [40] using the Monte Carlo algorithm and the Merck Molecular Force Field (MMFF) with standard parameters and convergence criteria. DFT and TDDFT calculations were run with Gaussian’16 [66] with default grids and convergence criteria. DFT geometry optimizations and frequency calculations were run at the ωB97X-D/def2-TZVP level of theory including the integral equation formalism of the polarizable continuum solvent model (IEF-PCM) for ethanol. TDDFT calculations were run at the ωB97X-D/def2-TZVP/PCM level of theory for the first 24 excited states. Calculated spectra were plotted as sums of Gaussians with 0.15 eV exponential half-width using the program SpecDis [67,68], using dipole-length rotational strengths. The results obtained with dipole-velocity rotational strengths were fully consistent. Vibronic absorption and ECD spectra were calculated at the same level of theory using a development version of the FCclasses code [52] and adopting the Vertical Hessian model with internal coordinates (VHint) on the first two excited states of each molecule. The vibronic line-shape was simulated adopting a time-dependent approach [69] with a Gaussian envelope having a half-width at half maximum (HWHM) of 0.03 eV. Average computational wall-times for each conformer (16-cores workstation, memory 24 Gb): ground state optimization/frequency, 2 h; TDDFT, 1.5 h; excited state frequency (analytical), 3.5 h.

## 5. Conclusions

We analyzed by means of DFT and TDDFT calculations the conformation and chiroptical (ECD) response of two structurally related 1,1-diarylcarbinols (**1** and **2**). These two compounds exhibit almost mirror-image ECD spectra in the region corresponding to benzene ^1^L_a_ and ^1^L_a_ bands, for the same absolute configuration at the carbinol center. Our calculations proved that the main discrepancy between **1** and **2** is a very different conformational situation. While compound **2** is more rigid, the absence of one *ortho* methyl group makes **1** more flexible and favors a different arrangement between the aromatic chromophores, which is the ultimate reason for the appearance of such diverse ECD spectra. We demonstrated that, in the case of compounds **1** and **2**, mirror-image ECD spectra arise—for the same absolute configuration—from a different conformation. Such a finding offers a good proof, which is immediately appreciable by students and non-experts, of the crucial impact that conformational factors may have on ECD spectra. More in general, all chiroptical properties result from an interplay of both configuration and molecular conformation, and should be analyzed and exploited consequently.

## Figures and Tables

**Table 1:** Low-energy conformers of compounds (*R*)-**1** and (*R*)-**2** calculated at ωB97X-D/def2-TZVP/PCM level with relative energies and populations.

	(*R*)-1			(*R*)-2		
Conf. ^1^	Energy ^2^	Pop. ^3^	*ϕ* ^4^	Energy ^2^	Pop. ^3^	*ϕ* ^4^
#1	0	41.0	72.5	0	52.3	−61.6
#2	0.139	32.4	74.5	0.366	28.3	−70.7
#3	0.748	11.7	−60.4	0.592	19.4	−60.8
#4	0.996	7.69	156.3			
#5	1.444	3.62	−66.8			
#6	1.451	3.59	−61.7			

^1^ Structures shown in Figure 2; ^2^ Internal energy in kcal/mol, relative to the absolute minimum for each compound; ^3^ Percent Boltzmann population at 300 K, estimated from internal energies; ^4^ Dihedral angle defined by carbon atoms 1″–1–1′–2′, in deg (indicated in Figure 2).

## Supplementary Materials

Supplementary materials are available online. Figure S1: Calculated weighted-average ECD spectra in the 185–260 nm range, Figure S2: Calculated weighted-average vibronic absorption spectra in the ^1^L_b_ region, Figures S3 and S4: Calculated ECD spectra for conformers #1-#6 of (*R*)-**1** and #1-#3 of (*R*)-**2**.
