# Supplementary material for: How and How Much Molecular Conformation Affects Electronic Circular Dichroism: The Case of 1,1-Diarylcarbinols
Source: Molecules. 2018 Jan 9;23(1):128. doi: 10.3390/molecules23010128 (PMC6017593; doi:10.3390/molecules23010128)
Supplement: Supplementary file 1 [file molecules-23-00128-s001.pdf]

Supplementary Material: How and How Much Molecular Conformation Affects Electronic Circular Dichroism: the Case of 1,1-Diarylcarbinols

Daniele Padula and Gennaro Pescitelli

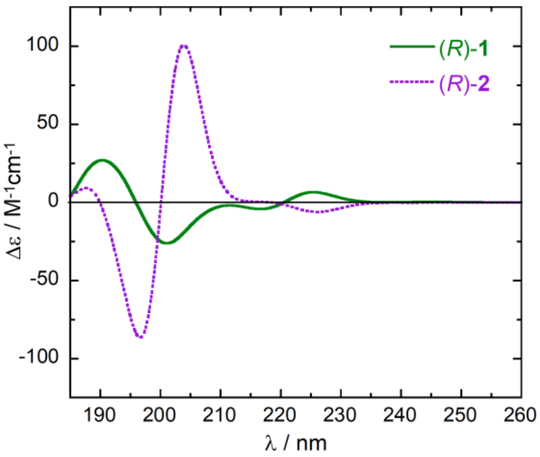

Figure S1. Calculated weighted-average ECD spectra of (*R*)-**1** and (*R*)-**2** at ωB97X-D/def2-TZVP/PCM level. Spectra plotted as sums of Gaussians with 0.15 eV exponential band-width and shifted by +15 nm.

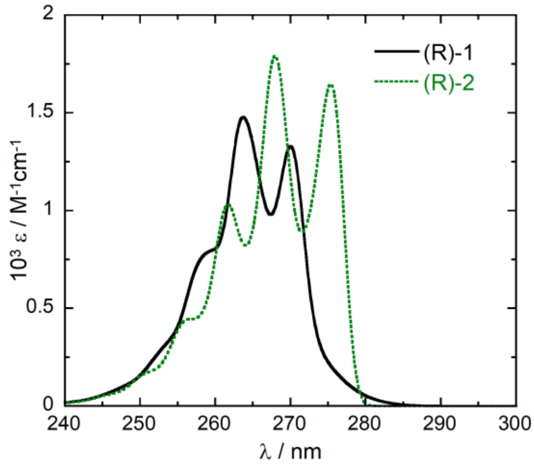

Figure S2. Calculated weighted-average vibronic absorption spectra of (*R*)-**1** and (*R*)-**2** in the ^1L_b region. Calculated spectra plotted as sums of Gaussians with 0.025 eV exponential band-width, shifted by +30 nm.

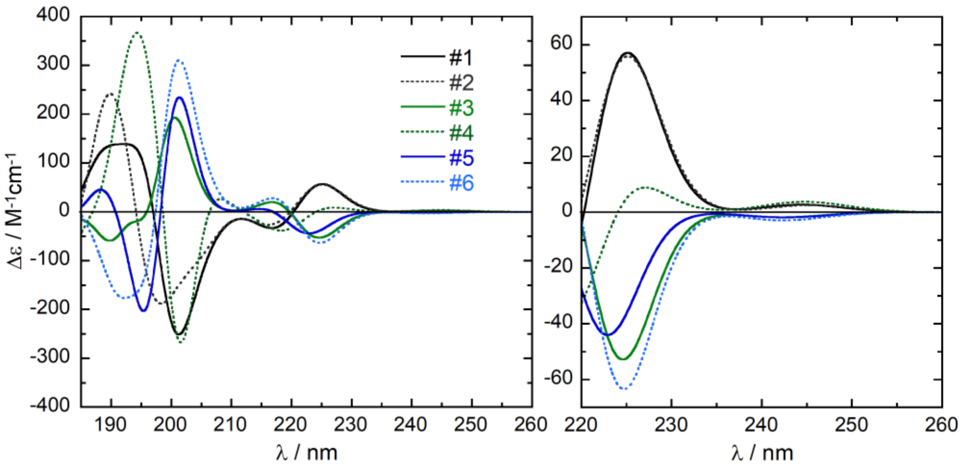

Figure S3. Calculated ECD spectra for conformers #1-#6 of (*R*)-**1** at ωB97X-D/def2-TZVP/PCM level in the 195-260 nm range. The plots on the right show an expansion of the ^1L_a/^1L_b region. Spectra plotted as sums of Gaussians with 0.15 eV exponential band-width and shifted by +15 nm.

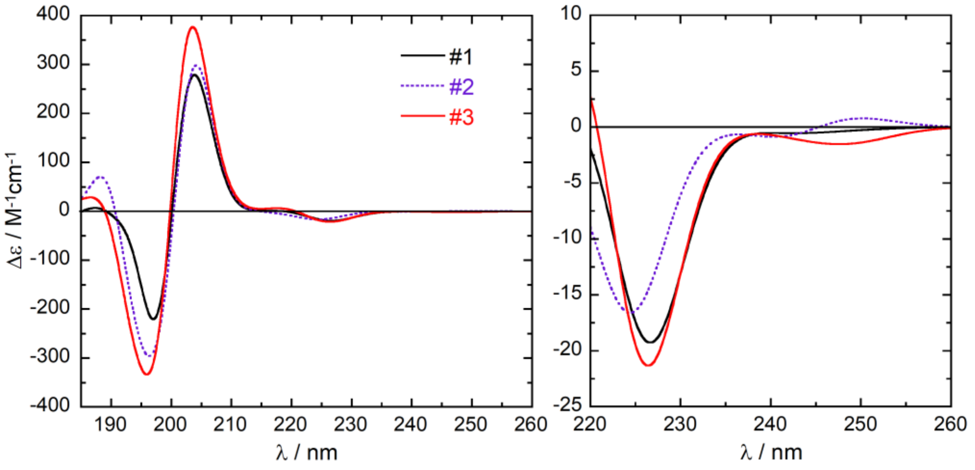

Figure S4. Calculated ECD spectra for conformers #1-#3 of (*R*)-**2** at ωB97X-D/def2-TZVP/PCM level. The plots on the right show an expansion of the ^1L_a/^1L_b region. Spectra plotted as sums of Gaussians with 0.15 eV exponential band-width and shifted by +15 nm.
